# Design and Development of a Family of Integrated Devices to Monitor Animal Movement in the Wild

**DOI:** 10.3390/s23073684

**Published:** 2023-04-02

**Authors:** Laila Daniela Kazimierski, Andrés Oliva Trevisan, Erika Kubisch, Karina Laneri, Nicolás Catalano

**Affiliations:** 1 Consejo Nacional de Investigaciones Científicas y Técnicas, Centro Atómico Bariloche (CONICET), Comisión Nacional de Energía Atómica (CNEA), San Carlos de Bariloche R8402AGP, Argentina; 2 Centro Atómico Bariloche, Comisión Nacional de Energía Atómica (CNEA), San Carlos de Bariloche R8402AGP, Argentina; 3 Instituto Balseiro, Universidad Nacional de Cuyo, Comisión Nacional de Energía Atómica (CNEA), San Carlos de Bariloche R8402AGP, Argentina; 4 Instituto de Investigaciones en Biodiversidad y Medioambiente, Consejo Nacional de Investigaciones Científicas y Técnicas (INIBIOMA, CONICET-Universidad Nacional del Comahue), San Carlos de Bariloche R8400AGP, Argentina

**Keywords:** animal movement, inertial sensors, GNSS position, low-power device, IoT, tortoise tracking

## Abstract

Monitoring the tortoise *Chelonoidis chilensis* in the wild, currently in a vulnerable state of conservation in southern Argentina, is essential to gather movement information to elaborate guidelines for the species preservation. We present here the electronic circuit design as well as the associated firmware for animal monitoring that was entirely designed by our interdisciplinary research team to allow the extension of device features in the future. Our development stands out for being a family of low-cost and low-power devices, that could be easily adaptable to other species and contexts. Each device is composed of a sub 1 GHz radiofrequency IoT-compatible transceiver, a global navigation satellite system (GNSS) receiver, a magnetometer, and temperature and inertial sensors. The device does not exceed 5% of the animal’s weight to avoid disturbance in their behavior. The board was designed to work as a monitoring device as well as a collecting data station and a tracker, by adding only small pieces of hardware. We performed field measurements to assess the autonomy and range of the radiofrequency link, as well as the power consumption and the associated positioning error. We report those values and discuss the device’s limitations and advantages. The weight of the PCB including battery and GNSS receiver is 44.9 g, its dimensions are 48.7 mm × 63.7 mm, and it has an autonomy that can vary between a week and a month, depending on the sampling rates of the sensors and the rate of the RF signal and that of the GNSS receiver. The characterization of the device parameters will favor the open use of this development by other research groups working on similar projects.

## 1. Introduction

Animal trajectories, behavior, and movement have been extensively studied throughout the years. The movement patterns are the result of the interplay between environmental resources and an individual’s energy needs, together with the interaction with conspecifics as well as with other species [1,2]. First observations were performed by researchers in the field, sometimes with the consequence of disrupting animal behavior, but more recently these can be carried out using automated systems, with sensors attached to the animals or deployed on the field [3,4]. Therein, several devices have been developed to monitor animal movement [3,5]. For instance, low-cost GPS loggers based on Arduino technology were assembled to monitor movement, home range, migration and interactions of Eastern box turtles (*Terrapene carolina*) in northwestern Ohio [6]. Another system, used on tortoises in semi-natural habitats, was based on a low-power programmable device with a radio communication system together with an accelerometer and GPS, and allowed researchers to detect the nest-digging activity of three species of Mediterranean tortoises: (*Testudo hermanni*, *Testudo graeca*, and *Testudo marginata*) [7,8]. After identifying characteristic patterns in the accelerometer signal, corresponding to the digging activity, the system transmitted the geographic coordinates of the tortoise to a remote control center in real time. This information allowed researchers to localize the tortoises’ eggs and protect them from predators [8].

Monitoring devices have many benefits when studying animal ecology and conservation [4,9]. However, in many countries, access to commercial monitoring devices is often too expensive. Furthermore, these devices are not usually versatile, in the sense that it is not always possible to make software or hardware modifications to adapt them to particular needs.

In this sense, our work was motivated by the need for our own development to monitor the terrestrial Chaco tortoise *Chelonoidis chilensis*, the southernmost continental tortoise in the world [10], whose natural history is poorly known [11,12,13,14]. This species inhabits the ecoregions of the dry Chaco, Monte plains, and plateaus [14,15] of Bolivia, and from the west of Paraguay to the north of the province of Chubut, in Argentina [12]. It is seriously affected by the advance of the agricultural frontier in the dry Chaco and by the extensive goat and cattle farming in southern sectors, becoming the most affected reptile in the illegal pet market in Argentina [16]. For those reasons, this tortoise was categorized as vulnerable nationally [16], as well as internationally (IUCN 2014, https://www.iucnredlist.org/species/9007/12949680) (accessed on 25 March 2023). In particular, the eggs of the southernmost populations have a very long incubation period under the ground that varies from 10 to 16 months which, together with the recent introduction of cattle and wild boars, makes these populations even more vulnerable [17].

This work presents the specification, development, construction, and characterization of a low-cost and low-power device specifically designed for animal monitoring. In particular, we present the device characterization at the tortoises’s study site, located about 20 km north of the city of San Antonio Oeste, Province of Río Negro, Argentina. The device consists of a global navigation satellite system (GNSS) receiver, a radio frequency transmitter, inertial sensors, a magnetometer, and a temperature sensor. The maximum weight of the device will be given by 5% of the weight of the animal so as not to disturb its behavior, and is around 50 g for the specific case of *C. chilensis*.

All the device specifications are available with an open source philosophy, so that other research groups can adapt this development to their needs according to the species to be monitored.

## 2. Monitoring System

Technological advances greatly contribute to our ability to address questions about the movement of different species: (i) why do they move? (ii) how do they move? (iii) when and where do they move? and (iv) what are the ecological and evolutionary consequences of the movement [18]? Tracking animal movement using global navigation satellite system (GNSS) technology is an increasingly popular method of studying animal ecology, behavior, and conservation [19]. The appearance of the global positioning system (GPS), inertial sensors, and long-distance communication modules have revolutionized the study of animals, allowing researchers to collect a large amount of information remotely: it is possible to know the range of movement, the map of trajectories, migration data, and to evaluate the impact that the fragmentation of space and the exploitation of resources could generate, among others [6]. The positioning information provided by GNSS can be complemented with a navigation module that contains different sensors that allow to asses the type of activity of the monitored individuals, including accelerometers, magnetometers, gyroscopes, and thermometers, among others. Our device contains this equipment and, for the design and implementation of the monitoring systems, we took into account the following basic aspects:

- Affordable cost: given that the number of animals to be monitored in order to have statistically significant results must be high, the challenge is to achieve a low-cost equipment.
- Low weight and size: the equipment tracks the movement of animals, so it must be light enough to avoid behavior disturbance and small enough not to hinder their movements.
- Autonomy: one of the main objectives in animal monitoring systems is to achieve autonomy to monitor their movements for as long as possible.
- Two-way RF communication: this is required for monitoring in real time and to be able to configure the device on the field.
- Compatibility: achieving compatibility with standard tracking equipment is important to reduce investment in supplies and people training.
- Flexible design: both the schematic circuit design and the firmware development are expected to be easily adaptable for their use with other species.

**Figure 1:**
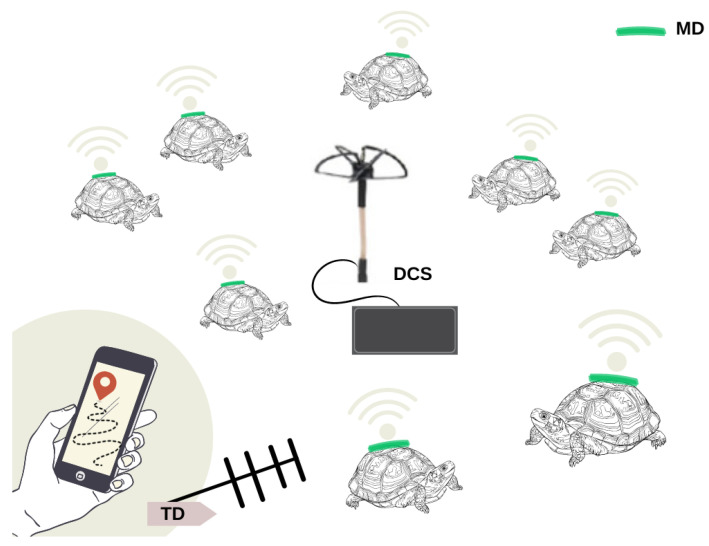
Schematic diagram showing the family of interacting devices: the monitoring device on individuals (MD), the data collector station (DCS), and the tracking device (TD).

Considering these characteristics we have developed a family of three devices: a monitoring device (MD), a tracking device (TD), and a data collection station (DCS), each of which will be explained below and its function is schematized in Figure 1. The three devices share the same schematic and printed circuit board (PCB) design.

The MD is responsible for collecting and storing the activity-related data of the animal under study. It is attached to the tortoise and acquires data from different sensors, i.e., an inertial measurement unit (IMU), a microphone, light, and temperature sensors, and a global navigation satellite system (GNSS) receiver. This information is stored locally and the MD periodically sends a subset of this data using radio frequency (RF)-modulated pulses. These pulses contain the GNSS position, animal activity level, and battery status, and are employed by the TD to retrieve the device after some time.

The MD periodically sends a subset of this data as messages using radio frequency (RF), using the Gaussian frequency shift keying (GFSK) modulation technique. These messages contain GPS position, animal activity level, and battery status, being received by the DCS. The MD also sends RF pulses every three seconds at a unique frequency for each MD to allow the MD to be tracked by the TD.

The TD is connected to a Yagi-Uda antenna that allows the detection of the direction of maximum received power to find the animal under study. The information of the received power is shown in an application running on a cell phone or tablet, through a Bluetooth interface. In this way, a researcher carrying a TD and a Yagi antenna uses the emitted RF pulses to track the position of a chosen MD. The task of the TD is not only to record animal movements but also to gather information about its surroundings, i.e., temperature and light conditions.

At the same time, the DCS collects all the received data, allowing the research team to monitor the status and location of the MD devices within the detection range, and in real time.

The DCS employs an omnidirectional circularly polarized antenna (to receive the pulse without preferential direction) to gather the information contained in the pulses sent by the monitoring devices.

Despite all three devices sharing the same PCB design, the selection of components allows us to reduce cost, power consumption, and size. In Table 1 we show the components required to build each device of the family.

The device must not exceed 5% of the animal’s weight and, in addition, it must be placed following specific care protocols. In particular, with tortoises, the protocol approved by the Institutional Committee for Care and Use of Laboratory or Experimental Animals (CICUAL) is respected, which includes techniques to reduce stress, hyperthermia, fluid loss, and disease transmission in order to guarantee the safety of the animals and their well-being.

To evaluate the performance of our development, we focused on autonomy, weight, size, and ease of modifying configuration parameters to be able to tune functionality for different applications. Regarding its size, we have a trade-off between making it as small as possible and letting us debug and characterize the design. We choose the components of this design in order to allow us to make in-house modifications if needed. Because of this, we choose a component package of 0805. In addition, the selection of the components was made in order to obtain the best availability and low-cost, autonomy, size, and weight ratio. It is important to notice that in order to strike a power-consumption we prioritize the selection of low-power components. In the case of the GNSS receiver, we take into account the ones available that came in a module presentation to allow an easy removal of the module in this first design.

### Components

When designing an electronic device there are certain essential factors to consider while making the selection of components. Among them we can mention: power consumption, sampling frequencies of the sensors, needed memory capacity, size, and market availability. With the main goal of reducing the design workload and simplifying components’ procurement, we decided to develop a PCB suitable for the three functionalities needed to study animal behavior, i.e., monitoring device, tracking device, and data-collection stations, as we already discussed. In the following paragraphs, we explain in detail the relevant characteristics of the chosen electronic components.

All three devices share the same microcontroller. In order to reduce both size and power we used the ultra low-power system on chip (SoC) CC1312R1 from Texas Instruments, Dallas, United States that integrates processing capacity and radio communication capabilities. This SoC contains three processors, the main one being an ARM Cortex M4F with support of machine learning Edge Impulse [20]. An independent core for controlling the sub 1 GHz radio (from 150 to 915 MHz) is compatible with the Internet of Things (IoT) and low-power wide-area network (LPWaN) standards such as SigFox [21]. It also contains an additional ARM R core for interacting with sensors at an extremely low-power consumption (31 μA at 2 MHz clock frequency).

To detect and to be able to recognize animal behavior we decided to employ inertial sensors, i.e., accelerometers and gyroscopes. We chose an ultra low-power nine-axis inertial measurement unit (IMU) from STMicroelectronics, Geneva, Switzerland. The device LSM9DS1 (https://www.st.com/en/mems-and-sensors/lsm9ds1.html (accessed on 25 March 2023)) features a three-axis accelerometer, gyroscope, and magnetometer, and a temperature sensor. This device has an internal FIFO buffer to store up to 32 samples for each sensor. It also allows to set up the threshold level of interruption for each sensor channel. This interruption can be used to wake up the microcontroller unit (MCU) from sleep mode and then resume normal operation. Combining this with different power-saving modes, supported by this IMU, we can make data acquisition more efficient in terms of power consumption and CPU usage.

We also included a microphone that may help to identify interaction with other individuals or to detect specific actions, such as eating or copulating. We chose the SPH0645LM4H-1-8 (https://www.knowles.com/docs/default-source/default-document-library/sph0645lm4h-1-datasheet.pdf (accessed on 25 March 2023)) solid state microphone from Knowles, Itasca, USA, due to its low power consumption (up to 600 μA), sleep mode, frequency range (at least 10 kHz), low cost, and software library availability.

For light-intensity sensing, we opted for the TSL2572 (https://ams.com/documents/20143/36005/TSL2572_DS000178_4-00.pdf (accessed on 25 March 2023)) from Osram, Premstaetten, Austria due to its ultra low-power mode (2.2 μA), wide measurement range (up to 600,000 lux), low cost, and software library availability.

The GNSS receiver NEO 7-M from Ublox was chosen because of its precision, price, and availability. We decided to employ a module in order to simplify the PCB design and because it allows us to change it without modifying the PCB design.

For data storage, we employed a micro SD memory card. This kind of card provides storage capacity over our needs, but at a very low price and with a convenient form factor and power consumption. They also allow us to download the data very easily to a computer.

The system also features a buck-boost DC–DC converter LTC3531 (https://www.analog.com/en/products/ltc3531.html (accessed on 25 March 2023)) from Analog Devices, Cambridge, United States, formerly Linear Technology, in other to obtain a 3.3 V output throughout the entire 3.7 V lithium polymer battery cycle (from 2.7 V to 4.2 V). For battery charging from a USB port, we selected the LTC4057ES5-4.2 (https://www.mouser.com/datasheet/2/609/4057f-2955004.pdf (accessed on 25 March 2023)) from Analog Devices. This also allows powering the system with a 5 V USB power bank for extended DCS operation and enhanced testing capabilities. Both components were selected due to their low size, ease of soldering, low pin count, and the minimal external components required. We chose to use LiPo batteries for our system due to their favorable balance between cost, energy capacity, size, and weight. These batteries have demonstrated acceptable performance over a lifespan of more than three summers of field work.

To lower the power consumption of the system, we employed the ultra low-power switch SiP32431 (https://www.vishay.com/docs/66597/sip32431.pdf (accessed on 25 March 2023)) from Vishay, Malvern, United States to allow a complete shutdown of both the micro SD memory card and the GNSS receiver. This switch is mandatory for the GNSS receiver due to the lack of an efficient power-saving function suitable for this application.

In addition to the mentioned components, the device features four LED indicators. One of them is connected directly to the USB port and turns on while connected. The other three indicate different situations depending on the device operation mode.

We employed three different kinds of antennas, each of them for a specific application. For the MD, we needed an antenna that does not disturb the animal behavior, so we opted for a wire antenna because it is lightweight and can be molded around the animal’s body.

In the case of the DCS, we employed an in-house developed omnidirectional circularly polarized antenna, known as cloverleaf [22]. This antenna receives signals from any direction and with any polarization. The third kind of antenna is the one employed in the TD. In this case, we used a linear polarized Yagi-Uda antenna because we wanted to detect the arrival direction of the signal. Since the antenna can be rotated by hand, polarization is not an issue so we can use a lighter antenna than the cloverleaf.

**Figure 2:**
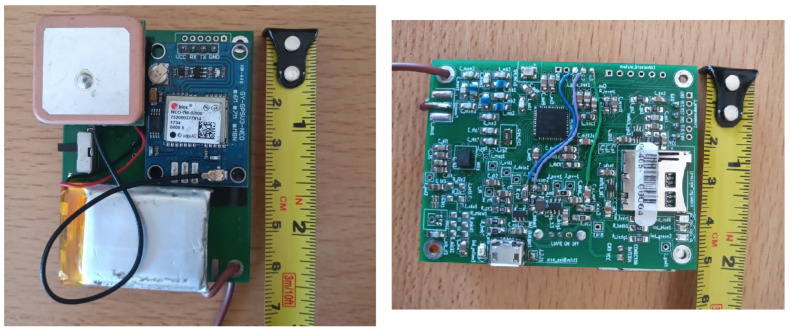
Both sides of the monitoring device (MD) with the corresponding components described in this section. The PCB board dimensions are 48.7 mm × 63.7 mm with 1.6 mm thickness. A ruler with a cm scale is included on the side.

The PCB with components for MD applications has dimensions of 48.7 mm × 63.7 mm and is 1.6 mm thick, and its weight is estimated at 9.2 g. The total weight of the PCB including battery and GNSS receiver, as shown in Figure 2 is 44.9 g, which represents approximately the 2.7% of the weight of a 1.5 kg tortoise.

In this case, the density of the material used is FR4 0.00185 g/mm${}^{3}$. A schematic of the circuit can be seen in the Supplementary Material.

## 3. Firmware

All functions of each device family member are controlled by a real-time operating system (RTOS) from Texas Instruments (TI-RTOS).

All implemented libraries were conceived following an encapsulatable, scalable, and flexible philosophy. For instance, in case of changing the MCU SoC, the encapsulation of hardware-dependent functions allows easy migration from one hardware platform to another. The design is scalable in the sense that allows the addition of tasks (such as sensors or processing algorithms) without modifying the design. It is also flexible, being able to modify dynamically and remotely the configuration of sensors, e.g., powering them up or down, lowering their sample rate to improve autonomy, or recording at a higher sample rate if needed. In this way, the same firmware architecture can be employed with the three device family members.

While the interaction between different elements of the system is mainly managed by the RTOS, the global scenario is controlled by a finite state machine (FSM). This FSM is responsible for the operation mode selection and controls the access to the micro SD memory card. The device has three modes of operation: charging, checking, and operational. Charging mode is entered when the device is connected to an USB port to charge its battery. In this mode, the MCU only monitors charging voltage and current. This information is useful to track battery behavior and determine its lifespan. Checking mode is the first state after powering on, when not connected to an USB port. While in this mode, the MCU checks for battery state, presence of micro SD memory card and every configured sensor, and waits until the GNSS receiver obtains a fix. In case any sensor check fails, an alert is indicated by LED flashing. While waiting for the GNSS receiver fix, another LED flashes. This device check, before attaching it to the animal in the field, allows ensuring the proper acquisition of the data over several days. Once all operational conditions are met, the device starts to gather data from all sensors according to each configuration, and stores it in the micro SD memory card. A subset of this data is periodically sent to the DCS over the RF link.

**Figure 3:**
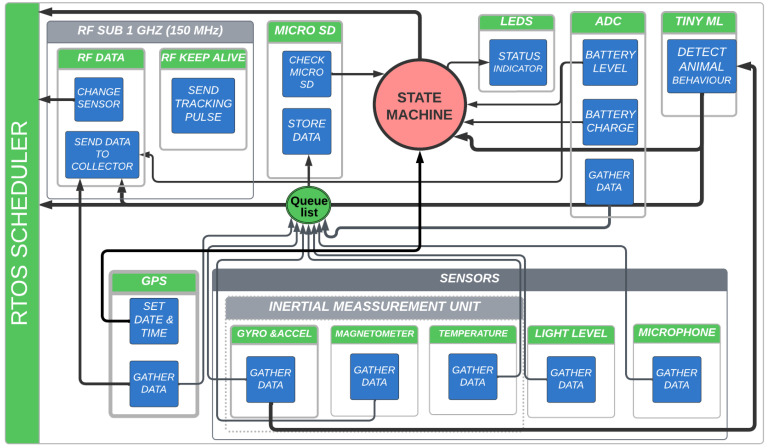
Firmware diagram with the different elements, actions, and tasks of the MD. Elements in light gray show examples of different drivers or objects that are being shared by tasks (such as the RF radio driver or the IMU). Blue squares represent different actions carried out by the RTOS tasks. Elements in green represent a single periodic/pseudo-periodic task being handled by the RTOS (TI-RTOS). The queue list for the micro SD memory card storage action is also managed by the OS. A state machine is an object called by the different tasks without being an autonomous task in itself.

The interaction between different elements of the system is mainly managed by the RTOS (see Figure 3). Each task (green) has its own structures attached, where data such as sample rate or sensor configuration is stored. The interaction between tasks is carried out with RTOS elements such as semaphores.

**Figure 4:**
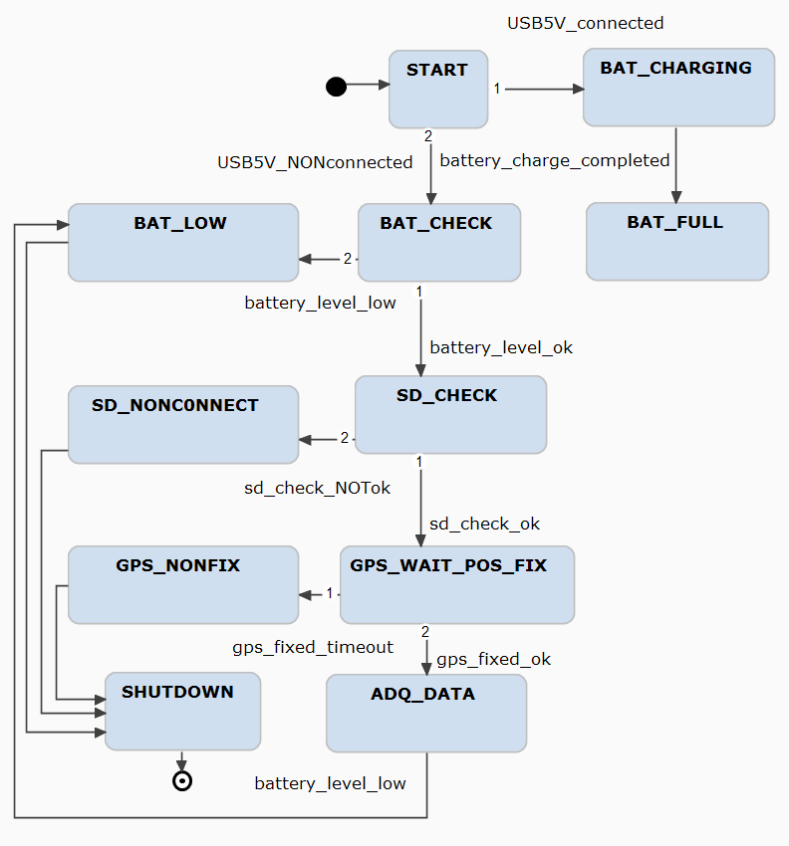
State machine reduced diagram created with Yakindu StateChart Tools (https://www.itemis.com/en/yakindu/state-machine/ (accessed on 25 March 2023)). It is operated by the different OS tasks and changes its behavior based on the current state.

The OS tasks interact with each other and with the FSM, as shown schematically in Figure 4. The FMS is in charge of processing the events and checks the state of the device. Based on the state, it orders the RTOS to carry out the corresponding actions, such as pausing sensors tasks to allow the system to go into sleep mode, and deleting or creating a new sensor task with a different configuration based on animal activity levels. All sensors gather data until their own buffer reaches a configurable level. When this happens, the task asks for permission to store the buffer content on the micro SD memory card, which is a shared resource among the OS tasks.

The TI-RTOS manages the tasks following a rate monotonic scheduling combined with the TI Drivers power-management policy. This allows the system to automatically change the power mode of the MCU or peripherals (such as the RF radio or the GNSS UART port), turning them into sleep mode until a new action is required to save energy.

## 4. Results

**Figure 5:**
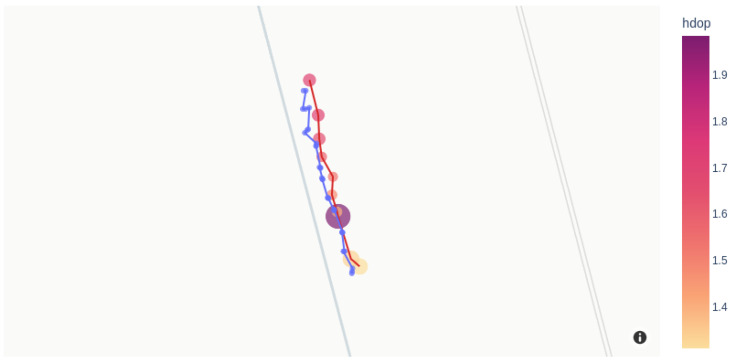
Positions measured with a Garmin GPS (blue circles) overlapped with the same positions measured with the GPS of the MD (yellow to red), at the tortoises’ study site. The radius of the circles indicates the measurement error assessed as explained in the text for each methodology. Hdop stands for horizontal dilution of precision.

In order to characterize the equipment, we measured several geographical positions over a straight line of 50 m every 5 m in a field near San Antonio Oeste inhabited by the tortoises, as shown in Figure 5. Each measurement was made with both a handheld Garmin eTrex 20 GPS receiver (blue dots) and the position data of the MD (red dots). The set of points measured with the handheld GPS represents the mean value of five measurements taken at the same position, being the radius of the circle and the standard deviation of those five measurements (blue dots).

However, the radii of the circles around the MD coordinate (shown in yellow to red in Figure 5) are proportional to the horizontal dilution of precision (hdop), which is an estimation of the GNSS measurement accuracy in latitude and longitude. This accuracy depends on the exact positions of the (usually four) satellites, relative to the GNSS receiver. For instance, a low hdop value represents better positional precision, and a value of hdop between one and two is considered accurate enough for most sensitive applications. These values are comparable to those reported in animal-tracking studies using GPS tracking technologies [23], although it is highly dependent on the geographical place where measurements were performed.

**Figure 6:**
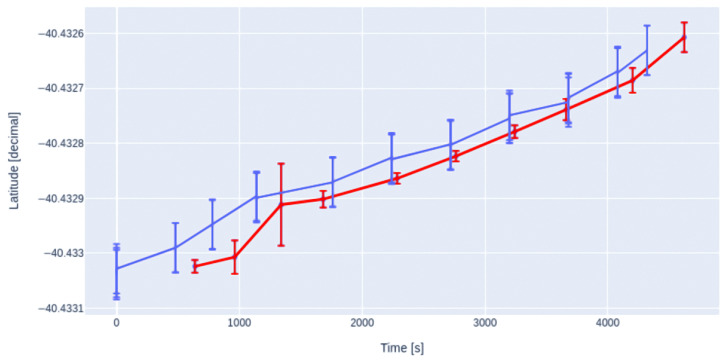
Latitude measured with a handheld Garmin GPS (blue) overlapped with the same positions measured with the MD (red), at the same positions plotted in Figure 5. The error bars represent the measurement errors as explained in the text for each methodology.

**Figure 7:**
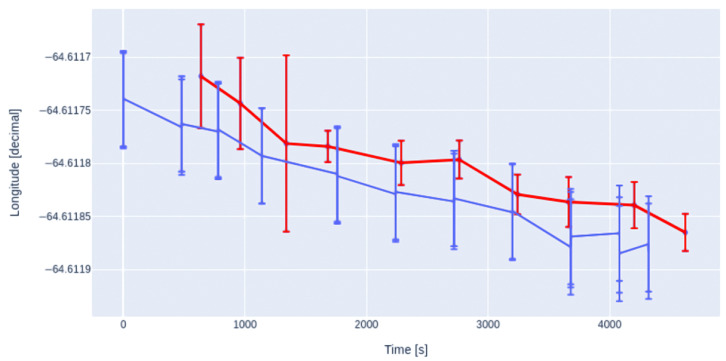
Longitude measured with a handheld Garmin GPS (blue) overlapped with the same positions measured with the GPS of the MD (red), at the same positions plotted in Figure 5. The error bars represent the measurement errors as explained in the text for each methodology.

As can be seen more clearly in Figure 6 and Figure 7, measurements taken with the handheld GPS are in good agreement with the geographical coordinates assessed by our device.

Regarding MD autonomy, we measured it in three different scenarios. All of these scenarios include a magnetometer sample rate of 10 Hz, temperature every 30 s, RF pulses with data sent each minute, and an RF tracking pulse every 3 s.

- Scenario 1: A week of autonomy, with the GNSS receiver taking position and hdop every 10 min (turning it off between samples) with the gyroscope and the accelerometer at 14.9 Hz. Here we are considering the use of a 1000 mAh battery and a trained machine learning model embedded in the device to determine if the tortoise has been moving or not, changing the turn-ON ratio of the GNSS receiver accordingly.
- Scenario 2: A minimum of 8.6 days of autonomy with gyroscope and accelerometer at 14.9 Hz.
- Scenario 3: A minimum of 26.7 days of autonomy with accelerometer at 10 Hz.

These values are of the order of the ones reported after tracking other species with similar sensors [24].

## 5. Discussion

Our device, completely designed by our team with an open-source philosophy, meets the requirements to monitor tortoises without disturbing their behavior. For instance, its low cost, appropriate weight and size, as well as good precision and autonomy are among those requirements. These characteristics outperform a previous Arduino prototype used by the research team in the field to gather movement information that allowed us to assess some basic quantities such as daily area visited by tortoises and trajectory characteristics [25]. With this new family of devices, we can monitor tortoises for several days with better accuracy. The autonomy of our design is compatible with the duration of the monitoring campaigns for this species. The agreement between the geographical positions recorded by our device and a handheld GPS encourages us to test the MD on tortoises in the wild.

By the analysis of the spectral characteristics of the accelerometer time series or simply by segmenting the signal according to a given relative intensity threshold, it is possible to detect tortoises’ movement and stillness [26]. Therefore it will be possible to make decisions in vivo to improve the autonomy of the equipment and the data acquisition, optimizing the software accordingly. In particular, the GNSS data-acquisition rate can be lowered when the tortoise is still, improving device autonomy. In addition, it was proved that some very relevant activities, such as digging a nest to lay eggs, can be detected in real time by the use of machine learning techniques [7,8]. Taking this into account, processor selection was carried out by looking for compatibility with machine learning capabilities. This feature will allow deploying on the device the algorithms that will perform the classification of behavior in real time, e.g., nest digging, copulating, or fighting. Additionally, the device can be configured to send a RF signal to the collection station when a given event is happening. For instance, if the event corresponds to eggs laying, we can implement protection measures to avoid eggs predation or trampling by cattle, which will be a very important step toward species conservation.

Furthermore, the MCU has an independent core for controlling the sub 1 GHz radio (from 150 to 915 MHz), compatible with Internet of Things (IoT) and Low-Power Wide area Network (LPWaN), both important to retrieve the activity of several tortoises simultaneously in real time. It also allows the implementation of a mesh network with the monitored tortoises if an increase of the monitored area is desired.

The low power consumption is essential in places where these tortoises live, without electricity and far away from urban centers. The flexible design of this family of devices will allow us to add other sensors, as well as to program other functionalities. For instance, the use of an added Bluetooth module to detect the proximity with other individuals without relaying over GNSS information, could help us to ascertain a better understanding of the social interactions among tortoises, a very little-studied aspect for reptiles in general.

Our devices have been proven successful in challenging environments, including dusty and rainy conditions. To ensure durability in such conditions, we encase them in a sealed container before mounting them onto animals. There is still work to be carried out for their possible implementation in other species, for example, addressing the challenge of making them smaller and lighter and, in addition, adapting them to underwater use.

We hope that the development of this family of devices will help other research groups working on the understanding of movement for the conservation of other vulnerable species.

## Figures and Tables

**Table 1:** List of components for each type of device of the family according to their functionalities. The "X" indicates the utilization of that component.

Elements	Monitoring Device (MD)	Tracking Device (TD)	Data Collection Station (DCS)
PCB	X	X	X
Sensors	X		
GNSS receiver	X		X
Bluetooth		X	
WiFi			X
Antenna type	“Wire”	Directional (Yagi)	Omnidirectional
Battery type	Rechargable LiPo 600 mAh	Rechargable LiPo 600 mAh	USB power bank 10,000 mAh

## Data Availability

Code and data are public and available at: git@github.com: TortoisesSAO/CodesDevice.git.

## Supplementary Materials

The following supporting information can be downloaded at: https://www.mdpi.com/article/10.3390/s23073684/s1. We include the schematic circuit of the monitoring device containing GPS, inertial sensors, temperature sensor and microphone using the CC1312R1F3RGZT microcontroller. We also show the results of the power consumption measured for each task of the device.
