# Supplementary material for: Design and Development of a Family of Integrated Devices to Monitor Animal Movement in the Wild
Source: Sensors (Basel). 2023 Apr 2;23(7):3684. doi: 10.3390/s23073684 (PMC10098901; doi:10.3390/s23073684)
Supplement: Supplementary file 1 [file sensors-23-03684-s001.zip › sensors-2182072-supplementary.pdf]

Supplementary Materials: Design and Development of a Family of Integrated Devices to Monitor Animal Movement in the Wild

In this supplement we will show the results of the power-constraint analysis, i.e. the power consumption measured for each task of the device:

- MCU: 100 uA with IMU, RF and GPS task. This value also depends on the sensors being used and their data rate (for example, a microphone requires by highers amounts of MCU activity in order to receive the data at a sample rate of 20 kHz and store it)
- RF data: 51 uA by sending minimum data (activity, battery level,position, temperature) each minute.
- RF tracking pulse: 0.1 mA at rate interval of 2.5 seconds between pulse.
- IMU: 2 mA with all instruments enabled at a rate of 14.9 Hz . 0.17 mA with only magnetometer and accelerometer enabled and 0.13 mA with only the accelerometer enabled.
- GPS NEO 7 M: 74 mA when attempting to fix position, which makes the power consumption of this part highly reliant on factors such as weather condition or animal position. Without a time limit to fix the position and shutdown the sensor if no position fix ever occurs , this consumption has probed to reach an average of 20 mA in the research field.
- MicroSD: 76 uA when writing 4096 bytes between a twenty seconds interval, being enough to store all the accelerometer and gyroscope data acquired at a sample rate of 14.9 Hz. This amount represents most of the stored data, and therefore, the power consumption of this task.
- Tiny ML: Estimated to 10 μA if used to classify between animal moving or still one time per second.

Most power consumption relies on the GNSS receiver, being an order of magnitude above all the others sensors while running full time. Besides the GNSS receiver, the gyroscope consumes more than all the other functions (MCU, accelerometers, magnetometer, radio and micro SD memory card) combined.

This device uses a CC1312R1F3RGZT microcontroller and comprises a GNSS receiver, inertial sensors, temperature sensors and a microphone. We include code examples for the IMU module transmitting and receiving data for this device. Using the first prototype that we developed for this device, we collected enough data to study the movement of the tortoise *C. chilensis*.

S1. Schematic circuit

Schematic circuit of the animal monitoring system containing GPS, inertial sensors, temperature sensor and microphone using the CC1312R1F3RGZT microcontroller.

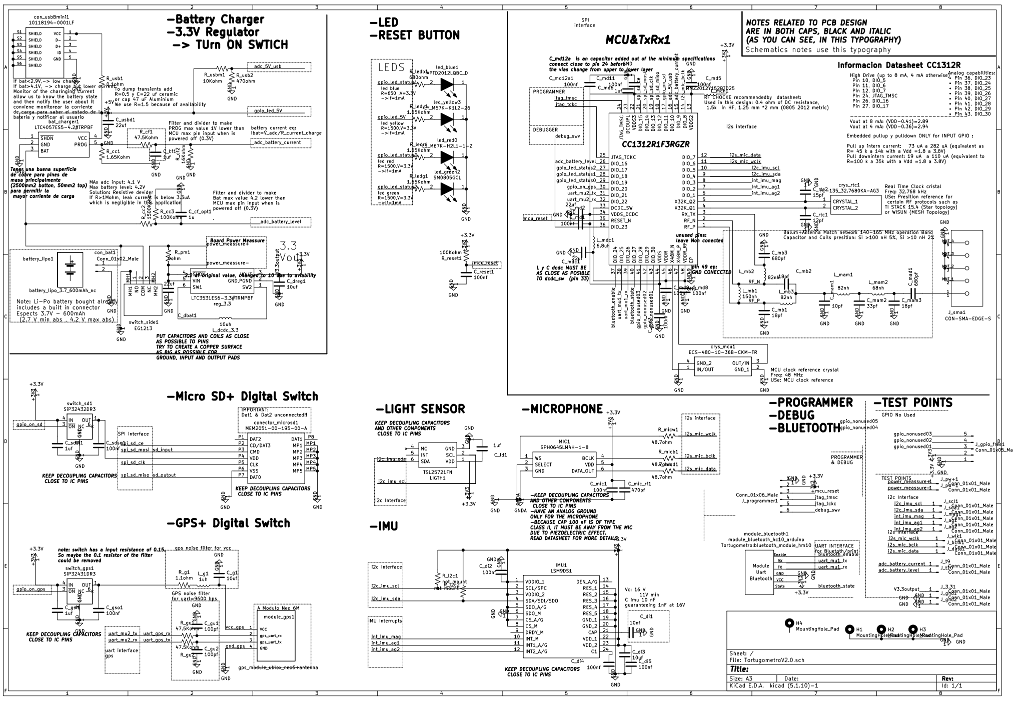

Figure S1. Schematic circuit of the monitoring device
